# Supplementary material for: Improving nonalcoholic fatty liver disease classification performance with latent diffusion models
Source: Sci Rep. 2023 Dec 7;13:21619. doi: 10.1038/s41598-023-48062-z (PMC10703886; doi:10.1038/s41598-023-48062-z)
Supplement: Supplementary file 1 — Supplementary Information. [file 41598_2023_48062_MOESM1_ESM.pdf]

Improving Nonalcoholic Fatty Liver Disease Classification Performance With Latent Diffusion Models

Romain Hardy[1,*], Joe Klepich[1], Ryan Mitchell[1], Steve Hall[1], Jericho Villareal[1], and Cornelia Ilin[1,†]

[1]School of Information, U.C. Berkeley

[*]First author
[†]Corresponding author: cornelia.ilin@berkeley.edu
[1]School of Information, U.C. Berkeley

November 28, 2023

Appendices

A Data Summary

A.1 Data Analysis

			Annotations: a = absent, p = present, pa = partial			Annotations: 0 = no, 0.5 = medium, 1 = high		
Patient_ID	NAFLD Class	Fat Content (%)	Cross-sectional image			Increased Hepatic Echogenicity	Blurred Hepatic Vein Borders	Blurred Diaphragm
			liver/kidney	hepatic veins	diaphragm			
1	0	3	p	p	p	0	0	0
2	0	4	p	p	a	0	0	n/a
3	0	2	p	p	a	0	0	n/a
4	0	1	p	p	p	0	0	0
5	0	2	p	p	p	0	0	0
6	0	2	p	p	a	0	0	n/a
7	0	2	p	p	a	0	0	n/a
8	0	2	p	p	pa	0	0	0
9	0	2	p	p	a	0	0	n/a
10	0	1	p	p	pa	0	0	0
11	0	1	p	p	pa	0	0	0
12	0	2	p	p	pa	0	0	0
13	0	3	p	p	a	0	0	n/a
14	0	3	p	p	pa	0	0	0
15	0	0	p	p	p	0	0	0
16	0	0	p	p	p	0	0	0
17	0	2	p	p	p	0	0	0
18	1	40	p	p	a	1	1	n/a
19	1	70	p	p	a	1	1	n/a
20	1	15	p	pa	a	0.5	0.5	n/a
21	1	7	p	p	a	0.5	1	n/a
22	1	70	p	p	a	1	1	n/a
23	1	25	p	p	a	1	1	n/a
24	1	20	p	p	a	1	1	n/a
25	1	10	p	p	pa	0.5	1	0.5
26	1	85	p	pa	a	1	0.5	n/a
27	1	75	p	p	a	1	1	n/a
28	1	55	p	p	a	1	1	n/a
29	1	20	p	p	pa	1	1	0.5
30	1	20	p	p	a	1	1	n/a
31	1	30	p	p	a	1	1	n/a
32	1	55	p	p	a	1	1	n/a
33	1	40	p	p	a	1	1	n/a
34	1	70	p	p	a	1	1	n/a
35	1	70	p	p	a	1	1	n/a
36	1	80	p	p	a	1	1	n/a
37	1	20	p	p	a	1	1	n/a
38	1	75	p	p	a	1	1	n/a
39	1	50	p	p	a	1	1	n/a
40	1	70	p	p	p	1	1	1
41	1	25	p	p	a	1	1	n/a
42	1	10	p	p	a	0.5	1	n/a
43	1	40	p	p	a	1	1	n/a
44	1	50	p	p	a	1	1	n/a
45	1	20	p	p	a	1	1	n/a
46	1	20	p	p	a	1	1	n/a
47	1	10	p	p	a	0.5	1	n/a
48	1	10	p	p	a	0.5	1	n/a
49	1	5	p	p	p	0.5	0.5	0.5
50	1	25	p	p	a	1	1	n/a
51	1	15	p	p	a	0.5	1	n/a
52	1	80	p	p	a	1	1	n/a
53	1	50	p	p	a	1	1	n/a
54	1	15	p	p	a	0.5	1	n/a
55	1	20	p	p	a	1	1	n/a

Supplementary Table 1: **Real ultrasound image analysis.** Columns 1-3 show the ID, disease label, and the percent fat content for each patient, as reported by Byra et al., 2018.[15] Columns 4-6 are binary indicators of the visibility of relevant anatomical structures, annotated by Dr. Joe Klepich. Specifically, these include the liver and kidney, hepatic veins, and diaphragm; "p" indicates that the structure is present, "pa" indicates that it is partially visible, and "a" indicates that it is absent. Columns 7-9 are binary indicators of three stylistic features commonly used to identify patients with NAFLD, also annotated by Dr. Klepich. These features include increased hepatic echogenicity, blurred hepatic vein borders, and a blurred diaphragm: 0 indicates the absence of the feature, 0.5 indicates that the feature is partially or weakly present, and 1 indicates that the feature is strongly present. Whenever an indicator cannot be evaluated for a patient, the corresponding cell is labeled "n/a."

A.2 Data Preprocessing

Although the preprocessing transformations are mostly the same for the diffusion and classification machine learning pipelines, there are slight differences between the two approaches. In the diffusion pipeline, image preprocessing is applied dynamically during training, thus if a raw image appears in n mini-batches it will generate n cropped and resized images. In the classification pipeline, the preprocessing is fixed beforehand, such that each raw image corresponds to a single preprocessed image. The preprocessed dataset is saved and reused across all classification experiments so that we can objectively evaluate the impact of synthetic images on model performance. Also, in keeping with the pretraining schemes of our CNN classifier backbones, each image is normalized using the mean and standard deviation of the ImageNet-1k dataset (Deng et al., 2009[33]).

B Methods Summary

B.1 Latent Diffusion Models

Latent diffusion models (LDMs) are variants of diffusion models that first encode images to a low-dimensional latent space representation using a pretrained encoder E, thus avoiding costly computations in pixel space. A visual diagram of a LDM is shown in Rombarch et al., 2021.[14] During training, an image x is fed through E to produce a latent vector z. The model then adds Gaussian noise to z according to a fixed Markov chain, producing z_T. The training objective of the model is to learn the reverse process, that is, how to denoise z_T back to its original state z. Mathematically, this objective can be expressed as:

$$L_{\text{LDM}} := \mathbb{E}_{E(x),\epsilon\sim\mathcal{N}(0,1),t}\left[\|\epsilon - \epsilon_\theta(z_t, t, \tau_\theta(y))\|_2^2\right], \tag{1}$$

where ϵ_θ is a denoising autoencoder (shown in the figure as a UNet model) and τ_θ is an encoder that feeds conditioning inputs y (i.e. text, semantic maps, and class labels) to ϵ_θ via concatenation and cross-attention layers. Finally, the recovered latent vector $\tilde{z}$ can be projected back to pixel space through the use of a pretrained decoder D.

C Supplementary Figures

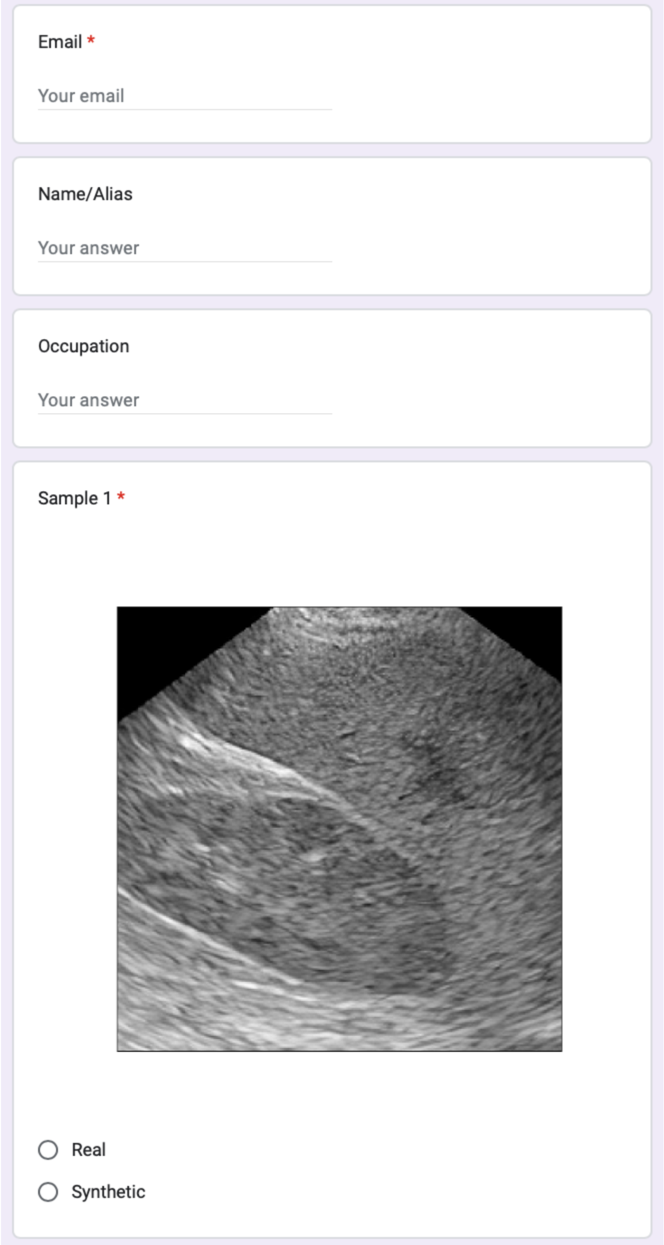

Supplementary Figure 1: **Turing test webpage.** A panel comprising five medical experts evaluated the realism ("Real" vs. "Synthetic") of 50 randomly selected images from our real and synthesized ultrasound databases. Image Sample 1 is shown here as an example.

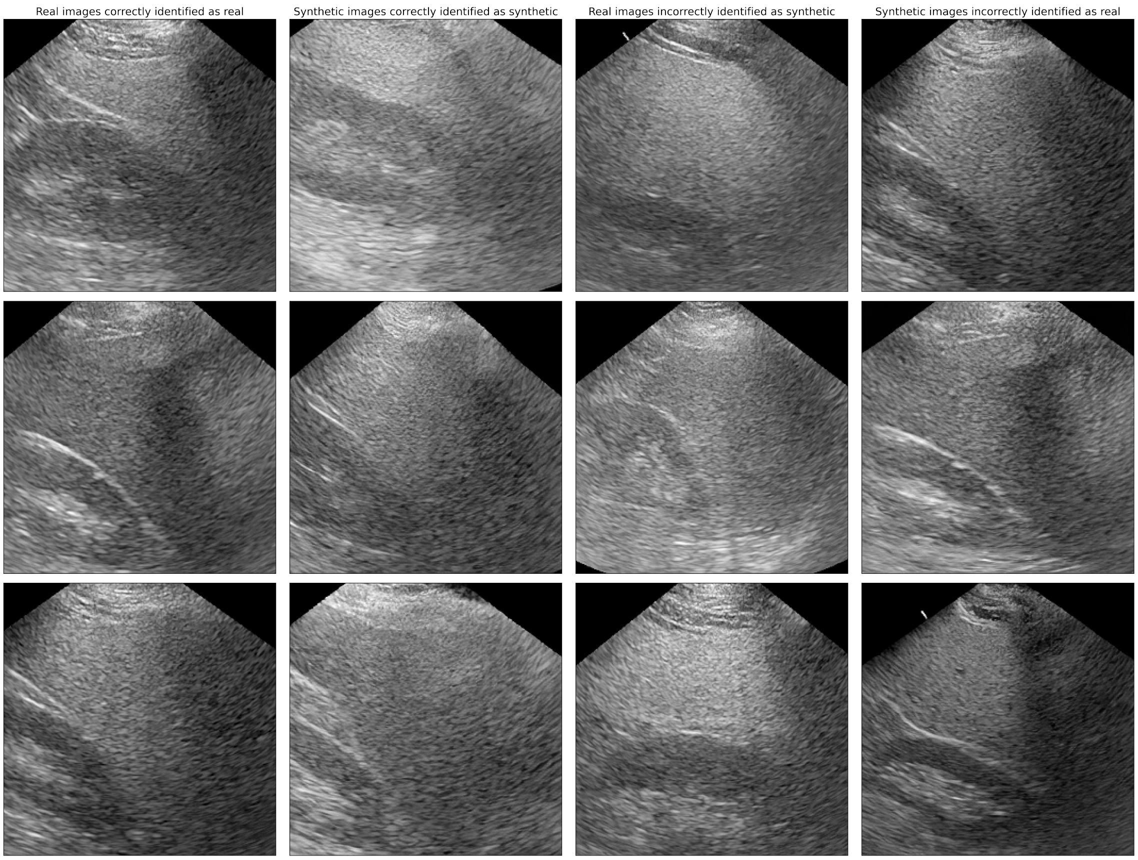

Supplementary Figure 2: **Top correct and incorrect samples from the Turing test.** The first two columns show the top three real and synthetic images most often correctly identified by test participants. The third and fourth columns show the top three real and synthetic images most often incorrectly identified by test participants.

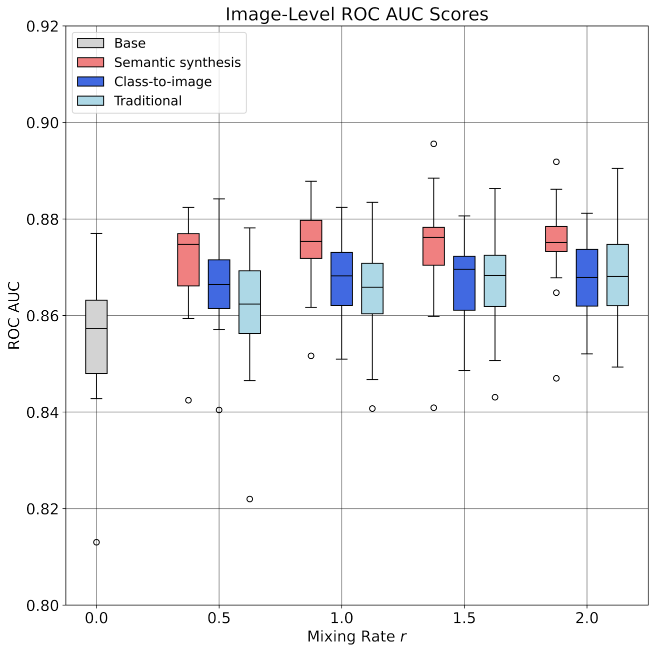

Supplementary Figure 3: **Image-level out-of-sample NAFLD classification performance – Sensitivity test 1.** These results are generated using the same ResNet-50 model as in Figure 5A. However, instead of concatenating the out-of-fold predictions and computing the ROC AUC, we average the ROC AUC across the five folds to produce a final fold-averaged score.

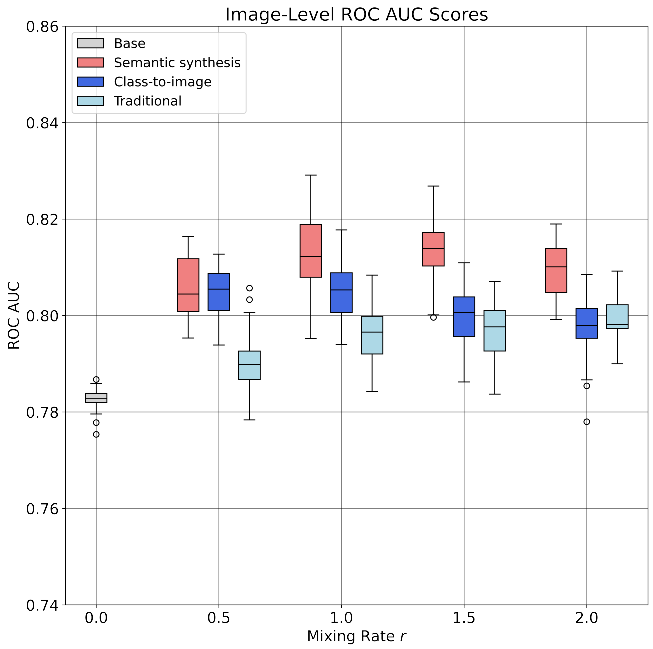

Supplementary Figure 4: **Image-level out-of-sample NAFLD classification performance – Sensitivity test 2.** This plot shows results for the same data input scenarios as in Figure 5A, except that we freeze every hidden layer in the ResNet-50 backbone.

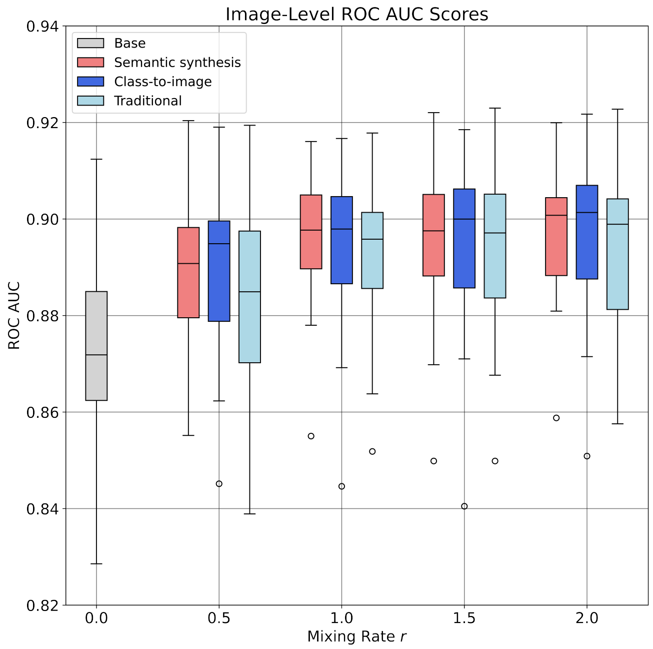

Supplementary Figure 5: **Image-level out-of-sample NAFLD classification performance – Sensitivity test 3.** This plot shows results for the same data input scenarios as in Figure 5A, except that we unfreeze every hidden layer in the ResNet-50 backbone.

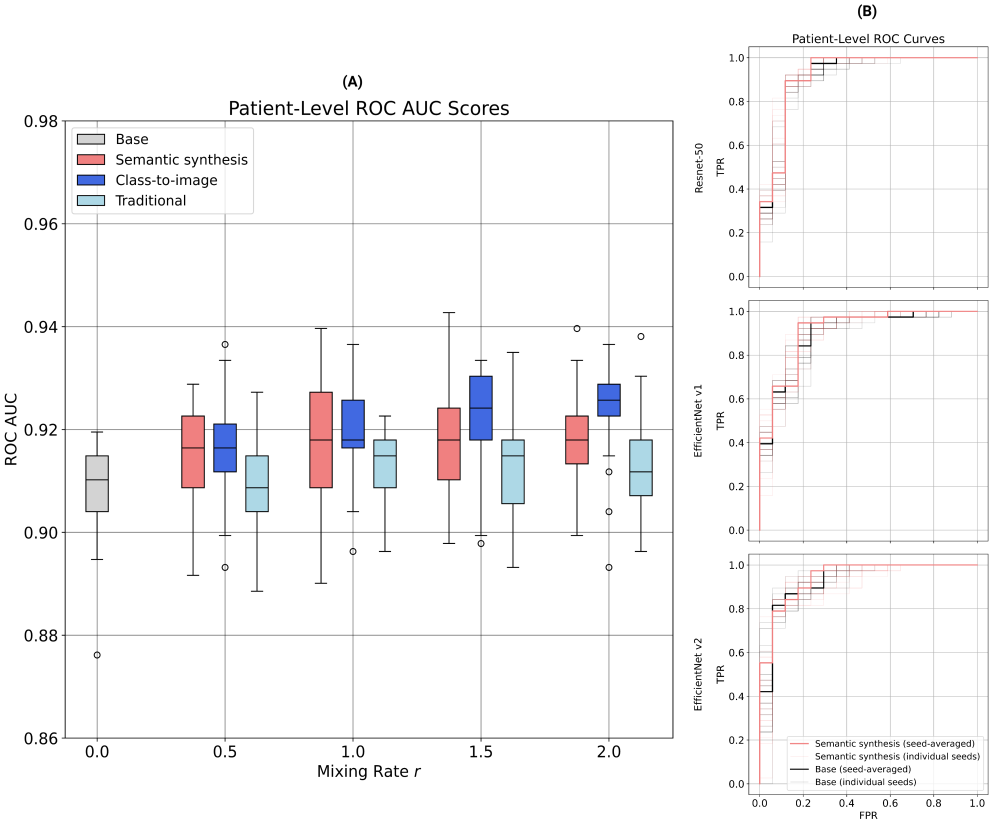

Supplementary Figure 6: **Patient-level out-of-sample NAFLD classification performance – Sensitivity test 4**. **(A)** Box plots of the patient-level ROC AUC as a function of the mixing rate r. **(B)** Patient-level ROC curves for three families of CNN classifiers: ResNet-50 (top), EfficientNet v1 (middle), and EfficientNet v2 (bottom). The average ROC AUC for the base and synthetically augmented models are as follows: ResNet-50, 0.907 versus 0.918; EfficientNet v1, 0.896 versus 0.908; EfficientNet v2, 0.932 versus 0.927.

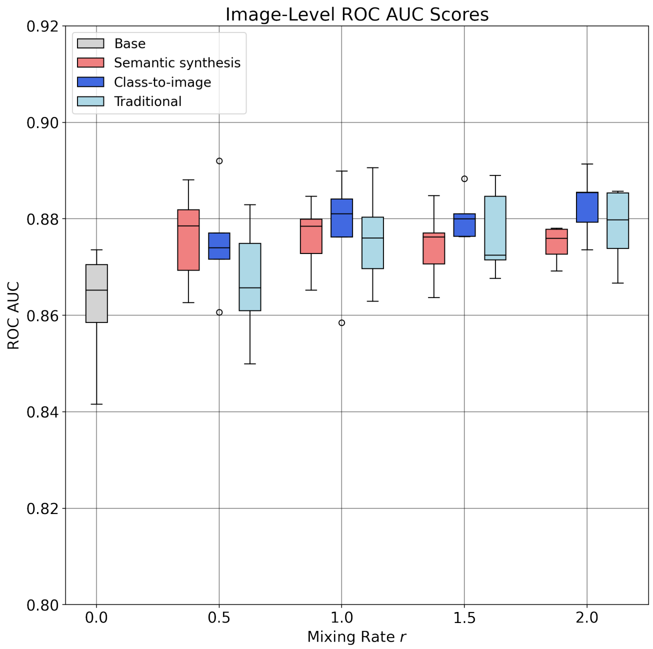

Supplementary Figure 7: **Image-level out-of-sample NAFLD classification performance – Sensitivity test 5**. Image-level ROC AUC as a function of the mixing rate r for five random seeds using balanced classes. Data balancing is done by upsampling the minority (healthy) class.
